# The Effect of a Newly Developed Oat-Banana Fermented Beverage with a Beta-glucan Additive on *ldhL* Gene Expression in *Streptococcus thermophilus* T_K_M_3_ KKP 2030p

**DOI:** 10.1007/s00284-016-1126-5

**Published:** 2016-08-26

**Authors:** Anna Goncerzewicz, Anna Misiewicz, Lubomiła Owczarek, Urszula Jasińska, Sylwia Skąpska

**Affiliations:** 1 Department of Microbiology, Culture Collection of Industrial Microorganisms, Prof. Wacław Dąbrowski Institute of Agricultural and Food Biotechnology, Rakowiecka 36, 02-532 Warsaw, Poland; 2 Department of Fruit and Vegetable Product Technology, Prof. Wacław Dąbrowski Institute of Agricultural and Food Biotechnology, Rakowiecka 36, 02-532 Warsaw, Poland

## Abstract

The number of people who cannot consume dairy products due to intolerance or allergies to food components is increasing. Consumers are increasingly searching for alternative, nondairy beverages which have an advantageous effect on the body and which stimulate gut microflora. Previous studies have shown that *Streptococcus thermophilus* T_K_M_3_ KKP 2030p can be an efficient starter culture for the fermentation of plant material and can also ensure an acceptable sensory profile and a high number of lactic acid bacteria during 4 week cold storage. The aim of this study was to determine the relative gene expression of the l-lactate dehydrogenase gene (*ldhL)* in selected strain using the RT qPCR technique at particular time points. The gene expression experiments were conducted in laboratory broth (LABm) and fruit–cereal (OBPromOat) matrix with a beta-glucan additive. Levels of gene relative expression in the selected strain in these two media were correlated with the amount of l-lactate produced. The results showed that the plant matrix stimulated stronger *ldhL* gene expression in the first 4 h of the experiment (although the synthesis of l-lactate was less than in laboratory broth). This study broadens existing knowledge of the transcriptional mechanisms which arise from the adaptation of the analyzed strain to different habitats during l-lactate synthesis. This could contribute to the development of new alternative food products for people who show intolerance to milk proteins or lactose.

**Electronic supplementary material:**

The online version of this article (doi:10.1007/s00284-016-1126-5) contains supplementary material, which is available to authorized users.

## Introduction

Scientific progress, particularly in the area of nutrition, is fundamental to the development of innovative food solutions. Undoubtedly, this has a huge influence on the improvement of key human body functions and, consequently, consumer health [30]. Recently, food companies have been making attempts to develop food products which offer multiple health benefits within a single food product [29]. Their task is not only to nourish the body, but preferably also impact upon its core functions; e.g., raise immunity, lower cholesterol levels, and protect against infections. It has been shown that consuming products based on bran and oats has positive effects on the body and can successfully replace dairy products for people who suffer from allergies. Oats and barley are major sources of beta-glucan, which is the main functional component of cereal fibers. In 1997, the Food and Drug Administration (FDA) officially acknowledged products made of whole-grain oats or oat fiber with a minimum of 0.75 g beta-glucan/serving size as being functional foods [30].

Previous studies have reported that, after appropriate processing, oats are a suitable substrate for fermentation with lactic acid bacteria and provide the foundation to create functional beverages [21, 22]. In order to improve the palatability and texture of the beverage, bananas have been chosen as an additional component.

Once created, such beverages contain high amounts of sucrose, glucose, and fructose, which have been hypothesized as potentially exhibiting good pro-health effects on consumers through stimulation of the growth of LABs, in parallel with suppression of enteropathogens in the colon, as well as through reduction of cholesterol levels in serum [11].

l-lactic acid can be directly involved in the human metabolism without any side effects. However, because mammalian tissues lack the enzyme metabolizing d-lactic acid, it has been reported that excessive intake of d-lactic acid may cause metabolic disorders. Therefore, fermented beverages containing lactic acid bacteria in l-lactate form only are recommended by the World Health Organization (WHO) for child nutrition, as well as the required 1 g of oat beta-glucan per beverage portion, thus complying with the EU requirement permitting an appropriate health claim on the label. Synthesis of l- or d-lactate varies among species of lactic acid bacteria. Some species can produce both d- and l-lactate as a result of racemase activity, or due to the presence of genetic determinants for both isoforms [25]. Many reports indicate that the genome of *S. thermophilus* lacks a functional *ldhD* gene and contain two separate l-lactate coding genes [5]. It has been indicated also that l-and d-lactic acid fall into two distinct enzyme families and there is no correlation between them in their process of evolution [16].

In our earlier research, we developed an oat-banana matrix fortified with a preparation of PromOat beta-glucan, which was successfully used as the nondairy base for a pro-health, sensorily highly acceptable, fermented beverage similar to yogurt. This product would have filled a gap on the market. A carefully selected LAB strain, *S. thermophilus* T_K_M_3_ KKP 2030p, was used, which ensured fast acidification of the matrix and a higher lactic acid bacteria population than in the beverage biotum is usually met in common fermented milk products [24].

The aim of the present study was to identify the one of two genes responsible for the production of l-lactate by *S. thermophilus* T_K_M_3_ KKP 2030p and to compare the relative gene expression in both the laboratory medium and the oat-banana matrix in which the fermentation occurred. Here, we compare the amount of metabolite produced with the relative gene expression at selected time points. Therefore, the point was investigated at which expression of the tested gene in selected media was induced. A further aim of this work is to investigate the transcription mechanism which regulates the production of l-lactate in *S. thermophilus* T_K_M_3_ KKP 2030p in a new fermented nondairy, yogurt-like beverage.

## Materials and Methods

### Strain, Media and Fermentation Conditions

A *Streptococcus thermophilus* T_K_M_3_ KKP 2030p strain stored in the Culture Collection of Industrial Micoorganisms of the Prof. Wacław Dąbrowski Institute of Agricultural and Food Biotechnology [32] was used in this study. This microorganism ensured both the best sensory profile for the fermented beverage and stability during 4 week cold storage [24]. The experiment employed laboratory broth without agar (LABm) [4, 9] and an oat-banana matrix with PromOat additive (OBPromOat). The additive containing 353 g kg^−1^ of beta-glucan was provided by BioVelop (Kimstad, Sweden) and prepared according to Owczarek [24]. The media were sterilized for 10 min at 118 °C, cooled to the fermentation temperature, and inoculated with 6 log of colony forming units ((cfu) g^−1^) of active culture. Fermentation trials were performed at 37 °C.

### Valuation of the Chemical Composition and Energy Content of the Oat-Banana Fermented Beverage with a Beta-Glucan Additive

Basic nutrients and energy contents were calculated based on the matrix formula and the average chemical composition of the primary ingredients [17], using the average conversion factors for following: proteins (17 kJ/4 kcal); fat (37 kJ/9 kcal); carbohydrates (17 kJ/4 kcal); and fiber (8 kJ/2 kcal) [28]. The dietary fiber and beta-glucan contents in the OB matrix were evaluated based on published data [23] and the producers’ certificates for beta-glucan preparations.

### Mono- and Disaccharide Quantification in the Developed Beverage

A single sample of the oat-banana beverage was diluted 1–4 and centrifuged for 10 min (6000 rpm). The quantification of mono- and disaccharide concentrations was carried out after filtration of the oat-banana matrix through a paper filter and then a 0.45 μm pore size membrane filter using a high-performance Waters 2695 liquid chromatography (HPLC) system (Waters, Milford, MA, USA) equipped with a Waters 2414 refractometric detector (Waters, Milford, MA, USA). A HPLC precolumn [Sugar-Pak and Guard-Pak (10 μm of particle size)] and a column [Sugar-Pak I (6.5 mm × 300 mm, 10 μm of particle size)] were used with 0.1 mM EDTA as the mobile phase under a temperature of 90 °C, elution flow rate was 0.5 ml min^−1^. 10 μl of a single sample was used each time.

### Sample Collection

Increases in lactic acid concentration in the oat-banana matrix and LABm broth are reflected in a drop in pH and acidification of the environment. The specific time points of 1, 4, 6, 7, and 24 h was chosen to analyze the relative expression of the *ldhL* gene.

### Acidifying Activity

The acidifying activity of the strain in the media was evaluated by pH measuring. OBPromOat matrix and LABm broth were inoculated with ca. 6 log cfu g^−1^ of 24-h culture, and incubated at 37 °C until pH was reduced to a level of about 4.0. The pH measurements were conducted with a Metler Toledo digital MP235 pH-meter at 20 °C. The fermented PromOat matrix and LABm broth were then stored at −20 °C.

### Determination of Lactic Acid

Total titratable acidity (TTA) was measured using AOAC method no. 2000.947.05 [1] and expressed as lactic acid (LA) (g kg^−1^). The content of l-LA and d-LA enantiomers was determined using an enzymatic kit (Cat. No. 11112821035, Roche Boehringer Mannheim Gmbh, Mannheim, Germany).

### Primers, PCR Reaction and Sequence Analysis

*Streptococcus thermophilus* T_K_M_3_ KKP 2030p DNA isolation was performed using the phenol–chloroform method according to Ausubel [2] and a commercially available Genomic Mini AX Bacteria Spin kit (A&A Biotechnology, Gdańsk, Poland).

The primers for *ldhL* gene identification used in this study were designed using Primer3 and Oligo 6.68 software based on a sequence (Accession No. D13405.1) from the NCBI-GenBank database. A new sequence for the examined strain was deposited in the GenBank database under accession no. LC056916. Based on the PCR, product primers for the *ldhL* gene for RT qPCR were designed. Furthermore, the *16S rRNA, gyrB, recA,* and *gyrA* genes were chosen as a potential candidate for reference, and appropriate primers were developed to obtain the required fragment length. The primers were purchased from Genomed S.A. (Warsaw, Poland). Each primer had previously been tested on 1.5 % (w/v) agarose gel to verify its specificity. Table 1 shows the primer sequences used in this study and the respective amplicon sizes.

**Table 1:** Genes and primers used for the PCR and RT qPCR experiments

Primer	Sequence (5′ → 3′)	Gene	Amplicon sizes (bp)	Application	Efficiency
l-dh_Strt_U	5′AAGTCATCCTTGTTGGTGACGG3′	*ldhL*	859	PCR	–
l-dh_Strt_L	5′TTCAATGGGATGTTTACTGGACG3′				
l-DH_RT_U2	5′ATTTGAAAAAGCCGTTGGTG3′	*ldhL*	102	RT qPCR	2.00
l-DH_RT_L2	5′GCATCCGCACAGTCTTCATA3′				
16STkM3_RTU	5′TTCTTGGATGAGTTGCGAACG3′	*16S rRNA*	74	RT qPCR	1.96
16STkM3_RTL	5′GTTTCCAATAGTTATCCCCCGC3′				
gyrB_RT_F	5′GCTCGCTATCACAAGTTGGT3′	*gyrB*	120	RT qPCR	1.96
gyrB_RT_R	5′GACATAGCCAGCTTCCAAGA3′				
recA_RT_F	5′AGCCATGCGTAAACTTTCTG3′	*recA*	119	RT qPCR	1.99
recA_RT_R	5′GTCCACCTGGGGTAGTCTCT3′				
gyrA_RT_F	5′CCCCTCAATACGTTTTTCCT3′	*gyrA*	100	RT qPCR	1.86
gyrB_RT_R	5′TCGTGAGCGCATTGTAGTTA3′				

Amplification reactions were performed in a 25 µl reaction volume containing 1 × reaction buffer (100 mM KCl, 100 mM (NH4)_2_SO_4_, 200 mM Tris–HCl (pH 8.5), 20 mM MgSO_4_, and 1 % Triton X-100), final concentrations of dNTPs (A&A Biotechnology, Gdańsk, Poland), and primers −0.2 pmol µl^−1^, 150–200 ng of DNA, and 1U of Run polymerase (A&A Biotechnology, Gdańsk, Poland). Amplifications were performed in an Eppendorf thermal cycler (Eppendorf, Germany). The reactions were run for 30 cycles with denaturation at 94 °C for 30 s, annealing at 56 °C for 30 s, and extension at 72 °C for 1:20 s. An initial 5-min denaturating step at 94 °C was used. *ldhL* gene sequencing was performed in Genomed S.A. (Genomed S.A., Warsaw, Poland).

### RNA Extraction and cDNA Synthesis

RNA extractions from the examined strain cultured in a LABm broth were performed using a commercially available kit [PureLink RNA Mini Kit, (Life Technologies, Grand Island, NY, USA)] with modifications and from an oat-banana matrix with the addition of TRIzol Reagent (Life Technologies, Grand Island, NY, USA) for difficult samples. Samples were ground to powder in a mortar under liquid nitrogen before adding the RNA extraction buffer. Genomic DNA was eliminated from the samples by DNase treatment according to the manufacturer’s description (RNase Free DNase Set, Qiagen, Hilden, Germany). RNA quality and concentration were assessed both spectrophotometrically to ensure an OD_260/280_ of 1.8 and higher (NanoDrop Technologies, Wilmington, DE, USA) and by electrophoresis in 1 % agarose. The RNA samples were preserved at −80 °C.

cDNA was synthesized and normalized to 50 ng µl^−1^ of total RNA and then diluted to the required amount using a RevertAid First Strand cDNA Synthesis Kit (Fermentas, Thermo Scientific, Waltham, MA, USA) with oligo (dT) priming. This was followed by RNase treatment according to the manufacturer’s instructions. The cDNA samples were stored at −80 °C.

### Expression Analysis of the *ldhL* Gene

RT qPCR analysis was performed in a Rotor Gene 6000 instrument (Qiagen) using double-stranded DNA-specific fluorochrome SYBR Green. Reactions were performed in a volume of 25 µl containing 1 µl of cDNA, 0.6 µl (10 µM) of forward and reverse primers, and 12.5 µl of 2 × SYBR Green master mix [Maxima SYBR Green qPCR Master Mix and Luminaris Color HiGreen qPCR Master Mix (Fermentas, Thermo Scientific, Waltham, MA, USA)] and DEPC (Sigma, Deisenhofen, Germany)-treated water.

The priming temperature and RT qPCR programs were based on thermal gradient tests. Twelve annealing temperatures were provided to ensure primer validation. To ensure primer specificity, agarose gels with the PCR product were run. The *ldhL* gene fragment for RT qPCR was also sequenced to exclude unspecific annealing of primers. This was performed in Genomed S.A. (Genomed S.A., Warsaw, Poland).

The RT qPCR reactions were run for 40 cycles with denaturation at 95 °C for 15 s, annealing at 57 °C for 30 s, and extension at 72 °C for 15 s. An initial 10 min denaturating step at 95 °C was used. In addition, product melting was assessed at the end of the reaction to verify the specificity of the reaction. Reactions conducted on the test samples gave Cq values that described the formation of the product, assuming a constant actual reaction yield. The threshold line was determined automatically by the software, and the Cq was uploaded to an Excel file for analysis using the double-delta method [19]. To test the efficiency of the reactions, standard curve were performed during all the runs using 10-point dilution series of a pooled cDNA sample. All experiments were performed with negative controls.

The *gyrB* and *recA* genes were selected as the reference on the basis of its stability in the tested samples. The most reliable reference gene for the assessment of relative gene expression was selected using BestKeeper 1.0 software that analyzed the standard deviation (SD) and coefficient of variance (CV) for replicate Cq values [26].

### Statistical Analysis

All determinations and experiments were performed in triplicate, and the results presented are the average values of three determinations. Statistica version 10 software from 2012 (StatSoft Poland, part of StatSoft Inc.) was used in the statistical evaluation of the experimental data. Two-way ANOVA analysis of variance with an LSD test (α = 0.05) was performed to evaluate the significant effect of time and medium (*P* < 0.05), as well as a significant interaction (time × medium) between these two variables (*P* < 0.05) in the expression of the examined gene.

## Results

### Nutritional Value and Saccharide Concentration in the Developed Beverage

The OB matrix evaluation was carried out in accordance with the principles given in the M&M section. The developed fruit–cereal beverage with a beta-glucan additive had a nutritive value approximately equal to its energy value −39 kcal and 164 kJ. It has been produced as a satisfying source of dietary fiber (11 g kg^−1^) and beta-glucan (5 g kg^−1^) for human consumption. Glucose, fructose, and sucrose concentrations in the OBPromOat matrix and in the developed beverage fermented with the selected strain are presented in Table 2.

**Table 2:** Mono- and disaccharide concentrations

	Sucrose (g kg^−1^)	Glucose (g kg^−1^)	Fructose (g kg^−1^)
OBPromOat matrix before fermentation	57.92 ± *0.10*	4.27 ± *0.02*	4.25 ± *0.01*
OBPromOat matrix after fermentation	52.16 ± *0.08*	3.94 ± *0.01*	5.36 ± *0.01*

### Acidifying Activity of the *S. thermophilus* Strain in Selected Media

**Fig. 1:**
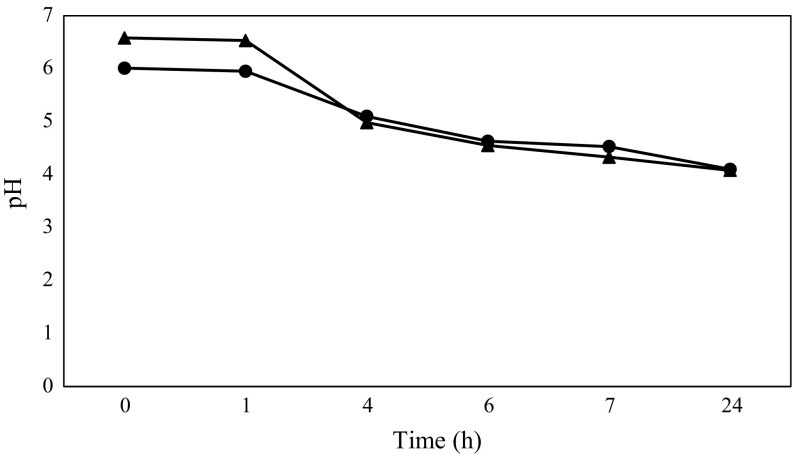
The acidifying activity of the *S. thermophilus* T_K_M_3_ KKP 2030p strain in (*filled circle*) OBPromOat matrix and (*filled triangle*) LABm broth

The acidifying activity of the *S. thermophilus* T_K_M_3_ KKP 2030p strain in different media was examined, and the results of the study can be seen in Fig. 1. The strain studied showed poorer growth in the oat-banana matrix than in the control media [24], but the pH value of the fermented product dropped in both media from 6.01 (OBPromOat) and 6.58 (LABm) to 4.1 similarly.

The study shows that, regardless of the environment, the analyzed strain had the same acidifying activity, which was demonstrated by the decrease in pH during the fermentation trials.

### Monitoring of l-Lactate Content

**Fig. 2:**
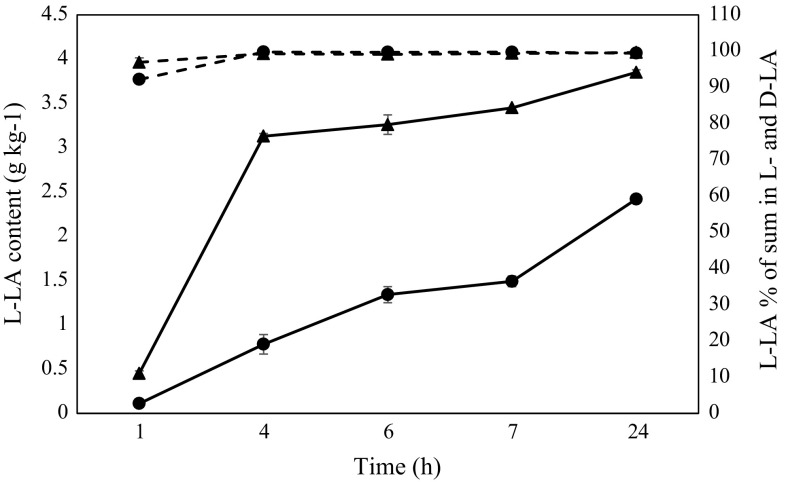
Changes in l-lactic acid enantiomer (l-LA) content (*solid line*) and percentage contribution of l-LA to the total LA content (*dotted line*) in (*filled circle*) OBPromOat matrix and (*filled triangle*) LABm broth under fermentation with the selected strain

During 24 h of fermentation, the content of l-LA enantiomers was measured, and it was shown that the selected strain produced larger amounts of this metabolite in laboratory broth (LABm) than in the OBPromOat matrix with a beta-glucan additive. The results are shown in Fig. 2. The final value of l-lactate in LABm broth was 3.85 ± 0.03 g kg^−1^, while in the oat-banana matrix, it amounted to only 2.42 ± 0.02 g kg^−1^ and contributed from 99.42 ± 0.54 to 99.62 ± 0.17 percent of the l-LA in the product’s total LA content. This amount was at minimal level and it was near the kit’s quantification threshold (specific data presented in supplementary materials).

The fastest increase in l-lactate in the LABm medium occurred during the first 4 h of fermentation and reached a level of 3.13 ± 0.03 g kg^−1^, which is identical to the rapid increase in the population of the examined organism in the medium (after 6 and 24 h of fermentation −8.28 ± 0.11 log cfu g^−1^). In the case of the strain cultured in the OBPromOat matrix, the amount of examined metabolite increased gradually and steadily, until it reached its highest level in the 24th hour of the experiment. These results correlate well with the slightly lower amount of cells grown in the medium (7.84 ± 0.01 g cfu g^−1^).

The high l-lactate content over d-lactate in the developed beverage plays a key role and is a valuable pro-health/functional component of the final product. In this study, the limitation of the d-lactate content in the beverage was successful due to the metabolic behavior of selected starters grown in the OBPromOat matrix.

### Selection of Reference Genes

We tested the transcriptional variability of four genes, previously reported in literature as potential reference genes: *16S rRNA*, *gyrB*, *recA,* and *gyrA*. SD (±Cq) and CV (% Cq) values were calculated for each of the candidate reference genes in the samples so as to identify the overall stability of gene expression. *16S rRNA* gene showed extremely high SD value and excessive distance of Cq value of the tested *ldhL* gene. Due to these facts, it cannot be taken to the further analysis. Second candidate *gyrA* showed an SD value of 1.79 (above 1), which also exclude its utility as a reference gene. The remaining two genes: *gyrB* and *recA* were selected as reference for gene expression studies, as the CV value for all replicates was 0.94 and 0.89, respectively. The parameters of the analyses are presented in Table 3.

**Table 3:** Descriptive statistics of candidate reference gene expression for all samples analyzed by BestKeeper 1.0

	*16S rRNA*	*gyrB*	*recA*	*gyrA*
N	17	22	19	17
GM [Cq]	9.08	23.96	25.20	13.02
AM [Cq]	10.41	23.99	25.22	13.23
Min [Cq]	2.12	21.21	21.56	10.54
Max [Cq]	18.62	25.73	26.40	19.87
SD [±Cq]	3.56	0.94	0.89	1.79
CV [% Cq]	34.24	3.93	3.54	13.50
Min [x-fold]	−108.46	−6.35	−12.21	−5.31
Max [x-fold]	612.27	3.30	2.29	100.32
SD [±x-fold]	11.00	1.89	1.82	3.32

*N* number of samples, *GM* [Cq] geometric mean of Cq, *AM* [Cq] arithmetic mean of Cq, *Min* [Cq] and *Max* [Cq] extreme values of Cq, *SD* [±Cq] standard deviation of the Cq, *CV* [% Cq] coefficient of variance expressed as a percentage on the Cq level, *Min* [x-fold], minimum value expressed as folds of expression, *Max* [x-fold] maximum value expressed as fold of expression, *SD* [±x-fold] standard deviation in fold of expression

### Identification and Expression of the *ldhL* Gene by RT qPCR

The PCR reaction demonstrated the expected product of the *ldhL* gene coding l-lactate dehydrogenases. After sequencing, the nucleotide sequence was deposited in the NCBI-GenBank database under accession no. LC056916.

The experiments involving the determination of *ldhL* gene expression in *Streptococcus thermophilus* T_K_M_3_ KKP 2030p were conducted in LABm broth and in an OBPromOat matrix. It was shown that the expression profiles of the analyzed gene differed statistically from each other depending on the components of the medium (*P* < 0.05). Time points did not have an impact on the experiment (*P* > 0.05).

In the case of LABm broth, the highest relative expression level was detected in the 6- and 7 h, and this was 2.3-fold higher than at the beginning. In the first time points, this level was low and constant; after 7 h, expression remained at an elevated level, while decrease to 1.03 in 24 h was observed.

**Fig. 3:**
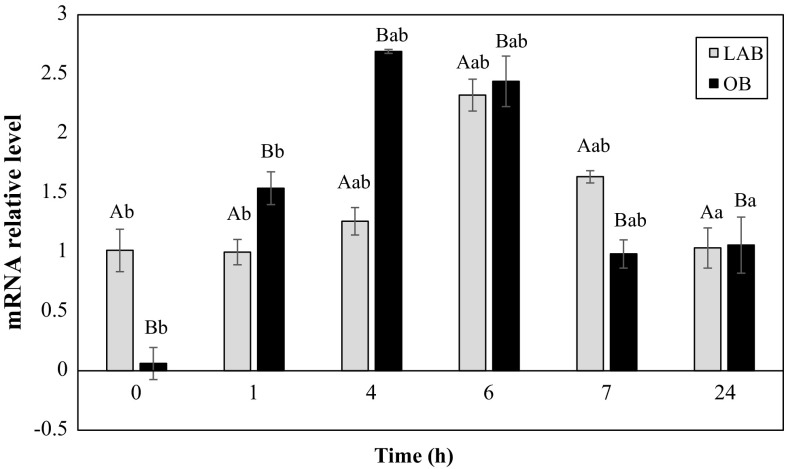
Lactate dehydrogenase (*ldhL*) gene expression for *S. thermophilus* T_K_M_3_ KKP 2030p strain during fermentation in (*black bars*) OBPromOat matrix and (*gray bars*) LABm medium. Different letters above bars represent significant effect of time and medium (*P* < 0.05), as well as a significant interaction (time × medium) between these two variables (*P* < 0.05). Factor “a” referred to 24-h; factor “b” referred to 0 and 1-h; factor “ab” referred to 4-, 6-, and 7-h. Factor A and B, referred, respectively, as medium LABm and OBPromOat

Different observations can be made in the case of OBPromOat matrix—the relative gene expression level was highest in the fourth and sixth hours of the experiment and then gradually decreased. The highest-fold change was indicated in the fourth hour, and this was 2 times higher than in LABm broth. Thereafter, it dropped to a value of 0.98 in the seventh hour. In the 24-h, there were no significant changes in the transcription level between OBPromOat and LABm medium. The results are shown in Fig. 3.

## Discussion

In recent years, the number of people who show intolerance or allergies to food components and thus cannot consume dairy products—which are a major source of beneficial intestinal microflora—has been increasing. Microorganisms which are present in the gut protect the digestive track from pathogens by producing lactic acid which provides them with the best environment for growth. As an alternative, vegetable, fruit, and cereal-based products have been developed, and these have become attractive to potential consumers, including children. Previously conducted research has shown that there is a possibility of creating an attractive sensory product, in addition to delivering valuable nutrients, which can stimulate the growth of beneficial gut microflora through the production of lactic acid [24]. In addition, supplying such a product in the soluble fraction of (1 → 3)(1 → 4)β-d-glucan, which acts as a prebiotic, can diminish hypercholesterolemic symptoms and reduce postprandial hyperglycemia [6]. It can also selectively stimulate the growth of bacteria which are beneficial for humans [23].

*Streptococcus thermophilus* is a dairy starter used in the production of industrial yogurt and is also linked with *Lactobacilli* strains. *L. delbrueckii* is a typical representative of d-LAB, because d-lactic acid accounts for over 80 % of its lactic acid products, whereas the l-lactic acid found in yogurt comes mainly from *S. thermophilus*. Ito and Sasaki [13] as one of the first characterized these bacteria in terms of their *ldhL* gene structure, primary sequence, gene expression, and protein structure using crystallography. l-lactic acid coded by this gene is one of the two lactic acid optical isomers and is produced via pyruvate from carbohydrates in diverse microorganisms and catalyzed by an NAD^+^-dependent l-lactate dehydrogenase [15]. During this reaction, NADH is used as a cofactor and plays an important role in over 700 biochemical reactions. Generally, l-lactic acid is the major form produced by lactic acid bacteria in the early-growth phase, whereas d-LA is formed in the late to stationary phase [3]. Using comparative genomics and high-throughput method for sequencing, it was shown that laboratory strains *S. thermophilus* CNRZ 1066 and LMG 18311 have two separate genes coding l-lactate dehydrogenase, which the first is placed between 606581 and 612349 bp and the second between 1129561 and 1132677 bp which may vary depending on the analyzed strain [5]. Comparative study demonstrate that analyzed region of *S. thermophilus* TkM3 KKP 2030p is placed between 1131721 and 1132539 bp and overlap with the location of second *ldhL* gene.

Our research shows that using *Streptococcus thermophilus* T_K_M_3_ KKP 2030p as a starter for OBPromOat fermentation ensures obtaining beverages which are characterized by a significant predominance of l- over d-lactate. Therefore, this approach is suitable for the production of nutritional products, as it conforms with the general recommendations of FAO/WHO [10] that limit the consumption of d-lactate to a max 100 mg per day for adults or that eliminate it from infant/child formulae. These recommendations stem from the recognized poor utilization of d-LA by children’s organisms and the risk of d-acidosis, particularly with children with short bowel syndrome and bacterial overgrowth [7].

The study shows that the selected strain grows faster in LABm broth than in the OBProm matrix with a beta-glucan addition due to the easier accessibility of the medium content. At the same time, the amount of l-lactate in the fruit–cereal matrix and laboratory medium fluctuated around the levels of 2.42–3.85 g kg^−1^, respectively. A similar observation was made by Mårtensson [22] for an oat-based, fermented beverage made with commercial yogurt cultures. However, it should be stressed that these fermented products could provide valuable sources of beta-glucans for consumers. Clinical trials have also demonstrated that the beta-glucooligomers of oat products have hypocholesterolemic and hypoglycemic effects [27]. Although the fact that beta-glucans can provide growth substrates—prebiotics [12] for some probiotic LABs and e.g., the intestinal strain *Clostridium difficile* [14]—here it became evident that *S. thermophilus* T_K_M_3_ KKP2030p was unable to utilize the beta-glucan of OBPromOat.

Our experiments show that the fruit–cereal medium with a beta-glucan additive stimulates relative expression of the examined gene in the first hours of fermentation, when the biosynthesis of l-lactate and logarithmic cell growth occurs. In the laboratory medium, expression of this gene is slightly higher in the sixth and seventh hour and remains at a constant level. In the LABm medium, the relative expression of the examined gene did not change significantly during the first hours of the fermentation trial, even when the cells are producing l-lactate. These results demonstrate that the environment significantly modulates the transcription apparatus of the cell. The ingredients of the OBPromOat matrix stimulate the reaction of the cells in increasing *ldhL* gene expression.

Some reports have indicated that environmental conditions have significant effects on LAB, and these are often linked to the related gene function. However, there are very few systematic studies about the molecular evolution of these two enzymes and the transcriptional analysis of *ldhL* and *ldhD* genes [18, 20, 32]. Cristescu et al. [8] examined the phylogenetic relationships of *ldhL* and –*D* genes during invertebrate evolution and suggested that the two enzymatic forms are not mutually exclusive. By analyzing the sequences and phylogenetic relationships of these genes, Zheng et al. [33] concluded that several nucleotide mutations in crucial catalytic sites probably cause different enzymatic activities, reflecting the variance of different types of LAB species in the evolution process.

Reports on various *Lactobacillus* strains have shown that the transcription levels of *ldhD* are higher than those of *ldhL* in representative LAB, i.e., from about 2- to 20-fold. There are no other obvious distinctions between *ldhL* and *ldhD* transcript levels among various *Lactobacillus* strains [33]. Transcription levels of *ldhL* and *ldhD* in *L. delbrueckii* ssp. *bulgaricus* DSM20081 (d-lactic acid producer) and *L. plantarum* ssp*. plantarum* DSM 20174 (dl-lactic producer) were comparatively investigated by Wang et al. [31]. In *ldhD*, transcription levels were higher than those of *ldhL* in both strains and similar to previously mentioned results. Unlike the situation in *Lactobacillus* strains, *ldhL* transcription in *Bacillus coagulans* was much higher than *ldhD* transcription in all growth phases, which may explain the high optical purity of the l-lactic acid produced. Although d-nLDH activities were not detected under native conditions, the transcription of the *ldhD*-encoding gene was detected by RT qPCR analysis [31].

The new possibility of making an acceptable, nondairy fermented product can be suggested in order to create new, alternative products where the transcriptional activity of lactate in applied strains would be successful. The indicated level of expression of the *ldhL* gene in the analyzed banana-oat matrix turned out to be promising and could have beneficial applications and be useful in the future.

## Electronic Supplementary Material

Below is the link to the electronic supplementary material.

Supplementary material 1 (DOCX 19 kb)

## References

[1] AOAC Official Method 2000.947.05 Acidity of milk. Tritimetric method. Gaithersburg, MD; USA: AOAC International

[2] Ausubel FM, Brent R, Kingston RE, Moore DD, Seidman JG, Smith JA, Struhl K (2001). Current Protocols in molecular biology.

[3] Axelsson L, Salminen S, von Wright A, Ouwehand A (2004). Lactic acid bacteria: classification and physiology. Lactic acid bacteria. Microbial and functional aspects.

[4] Bielecka M, Owczarek L, Grzybowski R, Majkowska A, Biedrzycka E, Markiewicz L (2002). Selection of lactic acid bacteria strains for fermentation of cereal and soybean preparations. Pol J Food Nutr Sci.

[5] Bolotin A, Quinquis B, Renault P, Sorokin A, Ehrlich SD, Kulakauskas S, Fonstein M (2004). Complete sequence and comparative genome analysis of the dairy bacterium *Streptococcus thermophilus*. Nat Biotechnol.

[6] Brennan CS, Cleary LJ (2005). The potential use of cereal (1 → 3,1 → 4)-β-d-glucans as functional food ingredients. J Cereal Sci.

[7] Connolly E, Abrahamsson T, Björkstén B (2005). Safety of D(-)-Lactic producing bacteria in the human infant. J Pediatr Gastr Nutr.

[8] Cristescu ME, Innes DJ, Stillman JH, Crease TJ (2008). d-and l-lactate dehydrogenases during invertebrate evolution. BMC Evol Biol.

[9] Davies JG, Ashton TR, McCaskill M (1971). Enumeration and viability of *L. bulgaricus* and *S. thermophilus* in yogurts. Dairy Ind.

[10] FAO/WHO (1974) Toxicological evaluation of certain food additives with a review of general principles and of specifications. In: WHO Technical Report Series (1–40). FAO and WHO, Geneva. http://dx.doi.org/10.1002/food.19860300707 4418402

[11] Fooks LJ, Fuller R, Gibson GR (1999). Prebiotics, probiotics and human gut microbiology. Int Dairy J.

[12] Gibson GR, Roberfroid MB (1995). Dietary modulation of human colonic microbiota: introducing the concept of prebiotics. J Nutr.

[13] Ito Y, Sasaki T (1994). Cloning and nucleotide sequencing of L-lactate dehydrogenase gene from *Streptococcus thermophilus* M-192. Biosci Biotechnol Biochem.

[14] Jaskari J, Kontula P, Siitonen A, Jousimies-Somer H, Mattila-Sandholm T, Poutanen K (1998). Oat β-glucan and xylan hydrolysates as selective substrates for *Bifidobacterium* and *Lactobacillus* strains. Appl Microbiol Biotechnol.

[15] John RP, Nampoothiri KM, Pandey A (2007). Fermentative production of lactic acid from biomass: an overview on process developments and future perspectives. Appl Microbiol Biotechnol.

[16] Kochhar S, Hunziker PE, Leong-Morgenthaler P, Hottinger H (1992). Evolutionary relationship of NAD+ -dependent d-lactate dehydrogenase: comparison of primary structure of 2-hydroxy acid dehydrogenases. Biochem Biophys Res Commun.

[17] Kunachowicz H, Nadolna I, Przygoda B, Iwanow K (1998). Food composition tables.

[18] Kylä-Nikkilä K, Hujanen M, Leisola M, Palva A (2000). Metabolic engineering of *Lactobacillus helveticus* CNRZ32 for production of pure l−(+)-lactic acid. Appl Environ Microbiol.

[19] Livak KJ, Schmittgen TD (2001). Analysis of relative gene expression data using real-time quantitative PCR and the 2(−Delta Delta C(T)). Methods.

[20] Llanos RM, Hillier AJ, Davidson BE (1992). Cloning, nucleotide sequence, expression, and chromosomal location of *ldh,* the gene encoding L-(+)-lactate dehydrogenase, from *Lactococcus lactis*. J Bacteriol.

[21] Marklinder I, Lönner C (1992). Fermentation properties of intestinal strains of *Lactobacillus*, of a sour dough and of a yoghurt starter culture in an oat-based nutritive solution. Food Microbiol.

[22] Mårtensson O, Andersson C, Andersson K, Öste R, Holst O (2001). Formulation of an oat-based fermented product and its comparison with yoghurt. J Sci Food Agric.

[23] Mälkki Y, Virtanen E (2001). Gastrointestinal effects of oat bran and oat gum—a review. LWT.

[24] Owczarek L, Jasińska UT, Skąpska S (2015). Development of oat-banana fermented beverage with beta-glucan additive. Pr. Inst. Lab. Bad Przem. Spoż.

[25] Pessione E (2012). Lactic acid bacteria contribution to gut microbiota complexity: lights and shadows. Front Cell Infect Microbiol.

[26] Pfaffl MW, Tichopad A, Prgomet C, Neuvians TP (2004). Determination of stable housekeeping genes, differentially regulated target genes and sample integrity: BestKeeper—Excel-based tool using pair-wise correlations. Biotechnol Lett.

[27] Rahar S, Swami G, Nagpal N, Nagpal MA, Singh GS (2011). Preparation, characterization, and biological properties of β-glucans. J Adv Pharm Technol Res.

[28] Regulation (EU) No. 1169/2011 of the European Parliament and of the Council on the provision of food information to the consumers. OJ L 304, sep 2011 p 18

[29] Sloan AE (2004). The top ten functional food trends. Food Technol.

[30] Verschuren PM (2002). Functional foods: scientific and global perspectives. Brit J Nutr.

[31] Wang L, Cai Y, Zhu L, Guo H, Yu B (2014). Major role of NAD-dependent lactate dehydrogenases in the production of l-lactic acid with high optical purity by the thermophile *Bacillus coagulans*. Appl Environ Microb.

[32] WDCM—World Data Centre for Microorganisms (WDCM 212) http://www.wfcc.info/ccinfo/index.php/collection/by_id/212/

[33] Zheng Z, Sheng B, Ma C, Zhang H, Gao C, Su F, Xu P (2012). Relative catalytic efficiency of ldhl-and ldhd-encoded products is crucial for optical purity of lactic acid produced by *Lactobacillus* strains. Appl Environ Microb.

