# Supplementary material for: The Effect of a Newly Developed Oat-Banana Fermented Beverage with a Beta-glucan Additive on ldhL Gene Expression in Streptococcus thermophilus TKM3 KKP 2030p
Source: Curr Microbiol. 2016 Aug 26;73(6):773–80. doi: 10.1007/s00284-016-1126-5 (PMC5059402; doi:10.1007/s00284-016-1126-5)
Supplement: Supplementary file 1 — Supplementary material 1 (DOCX 19 kb) [file 284_2016_1126_MOESM1_ESM.docx]

**Supplementary material**

Table 1 Growth of the *S. thermophilus* strain in the selected media

|  | Time (h) | | | | | |
| --- | --- | --- | --- | --- | --- | --- |
|  | 0 | 1 | 4 | 6 | 7 | 24 |
| LABm (log cfu g^-1^) | 6,00  ± *0.01* | 6.53  ± *0.08* | 8.28  ± *0,03* | 8.28  ± *0.11* | 8.28  ± *0.01* | 8.28  ± *0.02* |
| OBProm Oat (log cfu g^-1^) | 6.00  ± *0.01* | 6.46  ± *0.08* | 7.52  ± *0.05* | 7.72  ± *0.03* | 7.77  ± *0.01* | 7.83  ± *0.06* |

Table 2 Average Cq values of the *gyrB* and *recA* selected reference and *ldhL* target gene

| Medium | Gene | Time (h) | | | | | |
| --- | --- | --- | --- | --- | --- | --- | --- |
|  |  | 0 | 1 | 4 | 6 | 7 | 24 |
| LABm | *gyrB* | 24.37 | 23.88 | 21.53 | 22.59 | 23.27 | 23.42 |
|  | *recA* | 25.06 | 26.75 | 24.18 | 25.55 | 27.48 | 25.92 |
|  | *ldhL* | 21.19 | 21.81 | 19.01 | 19.35 | 21.16 | 21.12 |
| OBProm Oat | *gyrB* | 24.36 | 25.31 | 24.76 | 23.53 | 25.66 | 24.33 |
|  | *recA* | 26.36 | 26.28 | 25.70 | 24.23 | 24.51 | 24.92 |
|  | *ldhL* | 25.88 | 21.65 | 20.31 | 19.09 | 21.61 | 21.04 |

3. L-LA and D-LA content in OBProm Oat matrix and LABm broth under fermentation with the selected strain.

|  | Time (h) | | | | |
| --- | --- | --- | --- | --- | --- |
|  | 1 | 4 | 6 | 7 | 24 |
| **LABm broth** | | | | | |
| L(+) | 0.45  ±*0.03* | 3.13  ±*0.03* | 2.96  ±*0.04* | 3.45  ±*0.01* | 3.85  ±*0.03* |
| D(-) | 0.014  ±*0.005* | 0.024  ±*0.004* | 0.027  ±*0.001* | 0.011  ±*0.001* | 0.014  ±*0.007* |
| L-LA % of sum in L- and D-LA | 96.87  ±*1.23* | 99.24  ±*0.12* | 99.10  ±*0.01* | 99.69  ±*0.01* | 99.62  ±*0.17* |
| **OBProm Oat** | | | | | |
| L(+) | 0.11  ±*0.01* | 0.78  ±*0.11* | 1.34  ±*0.09* | 1.49  ±*0.06* | 2.42  ±*0.02* |
| D(-) | 0.009  ±*0.001* | 0.002  ±*0.003* | 0.004  ±*0.001* | 0.005  ±*0.007* | 0.014  ±*0.010* |
| L-LA % of sum in L- and D-LA | 92.15  ±*0.58* | 99.73  ±*0.38* | 99.67  ±*0.01* | 99.69  ±*0.43* | 99.42  ±*0.54* |
